# Children’s screen time and psychosocial symptoms at 5 years of age – the role of parental factors

**DOI:** 10.1186/s12887-024-04915-8

**Published:** 2024-08-03

**Authors:** Janette Niiranen, Olli Kiviruusu, Riitta Vornanen, Anneli Kylliäinen, Outi Saarenpää-Heikkilä, E. Juulia Paavonen

**Affiliations:** 1 Department of Public Health and Welfare, Finnish Institute for Health and Welfare, Helsinki, Finland; 2 Department of Social Sciences, University of Eastern Finland, Kuopio, Finland; 3 Faculty of Social Sciences, Psychology, Tampere University, Tampere, Finland; 4 Tampere Centre for Child Health Research, University of Tampere and, Tampere University Hospital, Tampere, Finland; 5 Pediatric Research Center, Child Psychiatry, University of Helsinki and Helsinki University Hospital, Helsinki, Finland; 6 Faculty of Social Sciences, Social Psychology, University of Helsinki, Helsinki, Finland

**Keywords:** Child, Screen time, Psychosocial symptoms, Child development, Parental mental health, Parenting styles

## Abstract

**Background and objectives:**

Electronic media (e-media) has become a universal part of young children’s daily lives. Previous studies have found an association between increased screen time and children’s psychosocial symptoms. We investigated whether parents’ psychological distress and parenting style dimensions explain the association between children’s screen time and psychosocial symptoms. Moreover, we investigated whether parents’ mental well-being and parenting style dimensions moderate this association.

**Methods:**

We used data from the Finnish CHILD-SLEEP birth cohort study. Parents and the child were assessed when the child was 5 years old (*N* = 671). The measure of screen time included program viewing from TV and other devices. Child’s psychosocial problems and parents’ depression, stress and parenting style dimensions were assessed by self-reports.

**Results:**

A high level of screen time in children was associated with attention and concentration difficulties, hyperactivity and impulsivity symptoms as well as internalizing and externalizing symptoms among 5-year-olds. For the most part, the associations remained significant despite controlling for parents’ mental health, parenting style dimensions and multiple background factors, especially associations relating to attention and concentration difficulties and hyperactivity symptoms were robust. Maternal stress and depression moderated the association between children’s screen time and psychosocial symptoms, indicating a more pronounced association among stressed or depressed mothers.

**Conclusion:**

There is an independent association between children’s screen time and psychosocial symptoms which is especially pronounced among those children whose mothers had poorer mental well-being. In clinical practice, the length of screen time should be inquired already at a young age and parents should be offered guidance to reduce the possible ill effects of excessive screen time, as well as help with their own mental health problems.

**Supplementary Information:**

The online version contains supplementary material available at 10.1186/s12887-024-04915-8.

## Introduction

Electronic media (e-media) has become a universal part of young children’s daily lives. For even younger children, e-media use is very popular sedentary behavior [1]. The American Academy of Pediatrics [2] (AAP) recommends limiting the use of e-media for children aged 2–5 years to one hour per day (see also the WHO [2]). Based on a recent study of Finnish 5-year-olds, the average daily screen time was almost two hours [3] and among American children aged 2–4 over two–and–a–half hours [4], thus considerably larger amounts than the limits recommended by the AAP. However, there are many potential risks associated with excessive e-media use at a young age [3, 5], such as increased internalizing [6, 7] as well as externalizing problems [6–10], including inattention [6–8] and impulsivity [6, 7]. In their longitudinal study, Christakis et al. [8] reported that hours of television viewing per day at both ages 1 and 3 years were associated with attentional problems at age 7 (OR = 1.09; 95% confidence interval [CI]: 1.03–1.15 and OR = 1.09; CI: 1.02–1.16]). Similarly, McNeill et al. [6] found that higher levels of program viewing at 3–5 years of age were significantly associated with increases in externalizing behaviors (β = 0.008; CI: 0.002–0.014) and total difficulties (β = 0.013; CI: 0.005–0.022) at 12 months later. Moreover, cognitive delays at preschool age have been reported among those children whose screen time was high during the early childhood years [11]. There seems to be a growing body of research on the potential harmful effects of screen time, but it is not yet clear how factors related to family functioning contribute to the effects of screen time in young children.

The factors related to family and it’s functioning are for example parents’ mental well-being and the parenting styles. They are potential factors that could modify the association between a child’s screen time and psychosocial symptoms. It has been reported that inconsistent parenting and inappropriate e-media content seems to add to the negative effects of screen time on low-income preschoolers’ executive functioning, while warm parenting and educational e-media content seems to decrease the negative effect of screen time and produce beneficial effects [12, 13]. Moreover, poor parent–child interaction seems to have an additional negative effect with increased screen time on preschooler’s psychosocial well-being [14]. Mistry et al. [15] noted that mothers who reported having a child who viewed screens for two hours or more were more likely to experience significant levels of depression. Moreover, when controlling for maternal depression, sustained television viewing was associated with negative behavioral outcomes, such as sleep problems, attention problems, aggressive behavior, and externalizing problems (according to the Child Behavior Checklist, CBCL). A study by Ansari et al. [16] pointed out how preschooler’s hyperactive behavior increased their screen viewing over time, and that the association was especially pronounced among those children whose parents were depressed and in families with lower socioeconomic status. All in all, it seems to be that the relation of parent’s mental well-being and parents’ stress as factors increasing screen time of young children have received only minimal research interest to date.

There is a long tradition of research on importance of parenting style for child development and well-being [17]. Parenting styles refer to the ways parents consider, respond and make demands in the interaction with their child; they describe patterns of behaviors and reflect attitudes and values parents have in their parenthood [18]. In contrast to the traditional parenting style paradigm that employs a typological approach (e.g., authoritative, authoritarian, and permissive parenting [19]), in the dimensional approach, parenting style characteristics are treated as continuous variables of a certain dimension [20]. The most studied parental style dimensions have been parental warmth and behavioral control [18], and the previous findings indicate the positive role of affective warmth as a parenting style on the effects of a child’s screen time [13]. Although there is evidence that some of the parenting style dimensions, such as parental warmth, play a role in the association between children’s increased screen time and psychosocial symptoms, the nature of the other parenting style dimensions remain unclear.

In the present study, our first aim was to examine whether parents’ psychological distress (parental depression and stress) or parenting style dimensions explain the association between children’s screen time and psychosocial symptoms, such as internalizing and externalizing symptoms. Second, we tested whether parents’ mental well-being and parenting style dimensions moderate this association. This study is a continuum from our previous study, where we reported associations with screentime and children’s internalizing and externalizing symptoms at the age of 5 years [3]. We expected that 1) screentime is an independent risk factor that adds to the well-established risks related to parental depression and stress and 2) some of the family-level risk factors moderate the risk related to screen time, so that the effect is especially pronounced when combined with a family-level risk factor, such as parental stress or depression.

## Methods

### Study design

This study is based on the five-year measurement point of the CHILD-SLEEP birth cohort. The recruitment and baseline measurement took place prenatally at the 32nd week and the follow-up measurements occurred at the birth of the child and at 3, 8, 18, 24, and 60 months of age. The study design, protocol, participants, and measures have been described in more detail in Paavonen et al. [21]. The study was approved by the by Pirkanmaa Hospital District Ethical Committee (9.3.2011, ethical research permission code R11032). The participants gave their written informed consent. Participation in the study was voluntary, and the families received no compensation for taking part.

### Participants

Mothers and fathers were recruited for the study from the Pirkanmaa Hospital District area in Southern Finland. Altogether, 2244 parents gave their approval to receive prenatal questionnaires when they visited the maternity clinics, and 1679 (74.8%) gave their consent to participate in the study and returned the baseline questionnaires. The response rate when the children were at 5 years of age was 42.5% (*N* = 714). Children with severe chronic illnesses or disabilities, e.g., Down’s syndrome or Hirschsprung disease (*n* = 7), and all twins (*n* = 8) were excluded. The final sample for the present study included those 671 children whose parents had responded to the Five-to-Fifteen (FTF) [22] questionnaire when their children were five. Regarding these 671 children, there were 455 fathers and 643 mothers who reported their mental well-being and parenting style dimensions when the child was 5 years of age. Background information and sociodemographic factors of the sample are presented in Table 1.

**Table 1:** Descriptive statistics of the study variables (*N* = 671)

Variable	Valid N	% (N) / Mean (SD)
**Sociodemographic factors**		
Child’s gender, girl	671	47.4 (318)
Child’s age, years; mean (SD)	670	5.68 (0.54)
University-level degree (at least one parent)	651	48.5 (316)
More than one child in household	649	85.7 (556)
Child in full-time daycare	634	67.8 (430)
**Child’s screen time**		
Program viewing at 5 years, min; mean (SD)	634	80.4 (36.3)
Over 60 min		66.8 (442)
Over 120 min		16.9 (112)
High dose (highest quartile; ≥ 88 min per day)		24.4 (157)
**Moderators, mothers**		
Depressed (CES-D ≥ 10)	644	21.1 (136)
Stressed (PSS-5 ≥ 11)	643	15.2 (98)
Parenting styles (CRPR)		
Low affection (lowest quartile)	644	23.3 (150)
Behavioral control (highest quartile)	644	25.2 (162)
Parenting stress (highest quartile)	644	23.8 (153)
**Moderators, fathers**		
Depressed (CES-D ≥ 10)	455	13.2 (60)
Stressed (PSS-5 ≥ 11)	455	9.9 (45)
Parenting styles (CRPR)		
Low affection (lowest quartile)	454	27.8 (126)
Behavioral control (highest quartile)	454	24.2 (110)
Parenting stress (highest quartile)	454	24.0 (109)
**Child psychosocial symptoms (FTF)**		
Attention and concentration; mean (SD)	668	3.76 (3.41)
Hyperactivity and impulsivity; mean (SD)	670	3.98 (3.63)
Internalizing; mean (SD)	670	1.52 (1.78)
Externalizing; mean (SD)	670	2.82 (3.25)

### Measures

#### Screen time

In this research the e-media use measure included screen time, i.e., program viewing on TV and other devices. Parents reported the time a child spent watching screens at 5 years of age. Separate questions were asked for weekday and weekend e-media use on how many hours a child watches programmes (including on television or other devices). For the analyses, we first recoded reported screen time into minutes. Second, we calculated a weighted daily average (5/7 on weekdays and 2/7 at weekends) of the measure (range 225 min). Finally, the measure was dichotomized using a 75-percentile cut-off to indicate those with the highest dose of e-media use: program viewing at 5 years of age for ≥ 88 min per day (24.3%, *n* = 155). The mean screentime in the high dose group (exceeding 88 min) was 131 min (SD 28 min, range 88–240 min) while it was 64 min in the low dose group (SD 19 min, range 15–88 min).

#### Parental depression (CES-D)

Mothers’ and fathers’ depressive symptoms were asked using the short 10-item version of the CES-D scale (The Center for Epidemiologic Studies Depression Scale) [23]. The scale included questions related to depressive symptoms, such as feeling lonely, feeling depressed, and restless sleep. After reversing two items, a higher sum score indicated more severe depressive symptoms (range 0–30 points). A cut-off score of ≥ 10 has been indicated to provide acceptable sensitivity and specificity against the criterion of caseness for clinically significant depression set by the original 20-item CES-D [24]. 

#### Parental stress

Stressfulness was measured using five items on a five-point scale derived from the Perceived Stress Scale [25] to gauge how unpredictable, uncontrollable, and overloaded the respondents find their lives to be (five items: “In the last month, have often have you felt that you were unable to control the important things in your life?” “In the last month, how often have you felt difficulties were piling up so high that you could not overcome them?”). The summary score (range 0–20) was dichotomized using a cut-off score of ≥ 11 (90th percentile) points to represent elevated levels of stress.

#### Parenting style dimensions

Mothers’ and fathers’ parenting style dimensions were measured using a Finnish version of Block’s Child Rearing Practices Report (CRPR) [26], which is a modernized version and assesses childrearing attitudes, values, and behaviors [18]. In the present study, we used affection, behavioral control, and parenting stress subscales of the measure. The affection subscale included items indicating a positive relationship with the child (10 items, e.g., “I often tell my child that I appreciate what they try out or achieve,” “I often show my child that I love them”). Behavioral control included items that showed how a child’s inappropriate behavior would have clear consequences and a parent’s willingness to confront a child who disobeys (six items; e.g., ‘‘My child needs to learn that we have rules in our family,” “When I am angry with my child, I let them know about it,”). Parenting stress included questions related to parents’ stress and guilt about their childrearing and parenting skills (Four items; e.g., “when I think of what kind of parent I am, I feel guilt or insufficiency,” “I have more problems with childrearing than I expected,”). In this study, to define the risk groups the subscales were dichotomized using the lowest quartile (to indicate low affection) or highest quartile (to indicate higher levels of behavioral control or parenting stress).

In this study, our aim was to study both stress in general and stress related to parenting. We have previously reported that maternal depression is related to children’s psychiatric symptoms at 2 and 5 years of age [27]. Later we also reported that paternal perceived stress is related to children’s psychosocial symptoms at 2 years of age [28]. Therefore, in this study both depression and perceived stress were considered in addition to parenting styles. Finally, we were specifically interested in stress related to parenting, as this might have been related to more screentime in children.

### Outcome measures

#### Child’s psychosocial symptoms (FTF)

The FTF questionnaire is an extensive questionnaire for parents about children’s developmental and emotional symptoms. It has been tested for its validity and reliability for the identification of internalizing and externalizing symptoms in children aged five to fifteen years [22, 29]. The questionnaire was developed to include Diagnostic Manual of Mental Disorders (DSM)-similar items of ADHD and of its most common comorbid condition in clinical practice. Other documented associated problem areas would also be covered in some detail, even though not necessarily covering all the symptoms of these conditions/problems as listed in the DSM [22, 29].

In this study, we used the following four subdomains: attention and concentration difficulties, hyperactivity and impulsivity, internalizing problems, and externalizing problems.

#### Background factors

Background factors that were controlled for in the analyses included the child’s gender (girl/boy), the child’s age, the parents’ education (at least one parent has a university-level degree, yes/no), whether there was more than one child in the household (yes/no), and whether the child participated in full-time daycare (yes/no).

Parental education was measured using an item with six response options (1 no vocational education, 2 vocational course/courses, 3 vocational school, 4 college or university of applied sciences 5 university, 6 other). This was categorized into two classes due to low prevalence of some classes (no education, vocational courses, other). Parental education was only used as a covariate in the model (i.e., it was treated as a confounding factor).

#### Attrition analyses

Attrition (nonresponse to FTF questionnaire at five years) was predicted by mothers’ depression (OR 1.48; 95% CI 1.06–2.05), stress (2.00; 1.30–3.10), lower than university level education (1.26; 1.02–1.55), previous children (1.38; 1.12–1.69) and younger age (0.96; 0.94–9.98) and father’s previous children (1.51; 1.22–1.86) in the prenatal measurement point.

#### Statistical methods

We first studied the distributions of the study variables and correlations between them. The continuous variables were reported using means and standard deviations, and dichotomous variables were reported as prevalence rates. Pearson correlation coefficients were calculated for the continuous variables. In the next stage, we made a series of ANOVA models to evaluate the influence of background factors and parental mental well-being as well as parenting style dimensions on children’s psychosocial symptoms, i.e., attention and concentration difficulties, hyperactivity and impulsivity, internalizing symptoms and externalizing symptoms.

The modeling was performed in five stages. First, we controlled only for the background factors: child’s gender, child’s age, parents’ education, number of children in the household, and child’s participation in full-time daycare. Next, separate models were analyzed for parent’s 1) depression, 2) stress, and 3) parenting style dimensions with all background factors in the model. In the last step, a model with background variables and all measures of parental distress and parenting style dimensions were simultaneously in the model. Separate models were analyzed for mothers and fathers. Before entering the variables to the multivariate models, multicollinearity was tested among the predictor variables using variance inflation factors (VIF) – all VIF values were < 1.4, thus indicating no signs of multicollinearity.

The moderating effects of parental distress and parenting style dimensions on the association between the child’s screen time and psychosocial symptoms were analyzed by adding interaction terms (child’s screen time x the parent-related variable in question) in the basic univariate model. In the next step we added background factors to the model. In supplementary analyses, moderation effects were analyzed using continuous measures of screen time and parental moderator variables (see Appendix 2; supplementary material).

## Results

Descriptive statistics of the sample are shown in Table 1. The mean age of the children in the sample was 5.7 years (standard deviation 0.5). The sample consisted of 318 girls (47.4%) and 353 boys (52.6%). The majority of the children (67.8%) were in full-time daycare. At least one parent had a university-level degree in 48.5% of the families. In 85.7% of the families, the children had siblings. Two-thirds (66.8%) of the children watched programs for > 60 min/day. We have reported the correlations between the main variables in the Appendix 1 (supplementary material). Mother’s mental health or parenting styles did not correlate with child’s screen time, except for maternal affection (*r* = -0.10, *p* < 0.05).

Among mothers, the first set of ANOVA models (Table 2, univariate models) showed that higher amounts of children’s screen time were related to their attention difficulties, hyperactivity, internalizing and externalizing symptoms, despite the background factors being controlled for (all ps ≤ 0.031). Except for internalizing symptoms, these associations remained significant when we controlled for maternal depression, stress or parenting style dimensions suggesting that screen time is related to psychosocial symptoms independent of parental distress and parenting style dimensions (Table 2, models 1–3). In the full model, with all the factors jointly studied, the associations of children’s screen time with attention difficulties, hyperactivity and impulsivity as well as externalizing symptoms remained significant (Table 2, models 4). Practically all (with only three exceptions) associations between measures of maternal distress and mother’s parenting style dimensions and the four child outcomes were significant when studied separately (univariate models), while mother’s parenting stress was the only factor that remained significant predictor of all four child psychosocial symptoms in the full models.

**Table 2:** The influence of mothers’ mental well-being and parenting style dimensions on the association between child’s screen time and psychosocial symptoms (Mother's *N*=722)

	Low risk group^a^	High risk group^a^	Univariate			Model 1^b^		Model 2^c^		Model 3^d^		Model 4^e^	
	Mean	Mean	B (se)	p	partial eta^2^	B (se)	p	B (se)	p	B (se)	p	B (se)	p
**FTF Attention and concentration difficulties**													
Screen time ≥ 75 pct	3.50	4.60	**1.10 (0.31)**	** < 0.001**	0.020	**0.93 (0.31)**	**0.002**	**1.04 (0.31)**	** < 0.001**	**0.95 (0.30)**	**0.002**	**0.92 (0.30)**	**0.002**
Depression ≥ 10 pts	3.45	5.10	**1.66 (0.33)**	** < 0.001**	0.039	**1.80 (0.32)**	** < 0.001**	–	–	**–**	**–**	**0.81 (0.37)**	**0.028**
Stress ≥ 11 pts	3.56	5.11	**1.54 (0.37)**	** < 0.001**	0.026	**–**	**–**	**1.74 (0.37)**	** < 0.001**	**–**	**–**	**0.81 (0.40)**	**0.043**
Parenting style dimension													
Low affection ≤ 25 pct	3.46	4.89	**1.43 (0.32)**	** < 0.001**	0.031	**–**	**–**	**–**	**–**	**0.82 (0.32)**	**0.011**	**0.80 (0.32)**	**0.013**
Behavioral control ≥ 75 pct	3.65	4.22	0.57 (0.31)	0.066	0.005	**–**	**–**	**–**	**–**	0.44 (0.30)	0.139	0.37 (0.30)	0.207
Parenting stress ≥ 75 pct	3.32	5.31	**1.99 (0.31)**	** < 0.001**	0.061	**–**	**–**	**–**	**–**	**1.82 (0.32)**	** < 0.001**	**1.36 (0.34)**	** < 0.001**
**FTF Hyperactivity and impulsivity symptoms**													
Screen time ≥ 75 pct	3.71	4.85	**1.14 (0.33)**	** < 0.001**	0.018	**1.05 (0.34)**	**0.002**	**1.12 (0.34)**	** < 0.001**	**1.06 (0.33)**	**0.002**	**1.04 (0.33)**	**0.002**
Depression ≥ 10 pts	3.72	5.10	**1.33 (0.35)**	** < 0.001**	0.022	**1.34 (0.36)**	** < 0.001**	–	–	–	–	0.56 (0.41)	0.169
Stress ≥ 11 pts	3.85	4.88	**1.04 (0.40)**	**0.010**	0.010	**–**	**–**	**1.24 (0.40)**	**0.002**	–	–	0.49 (0.45)	0.273
Parenting style dimension													
Low affection ≤ 25 pct	3.77	4.78	**1.01 (0.34)**	**0.003**	0.014	**–**	**–**	**–**	**–**	0.45 (0.36)	0.212	0.44 (0.36)	0.225
Behavioral control ≥ 75 pct	3.92	4.24	0.31 (0.33)	0.347	0.001	**–**	**–**	**–**	**–**	0.31 (0.33)	0.343	0.27 (0.33)	0.413
Parenting stress ≥ 75 pct	3.60	5.28	**1.68 (0.33)**	** < 0.001**	0.038	**–**	**–**	**–**	**–**	**1.58 (0.36)**	** < 0.001**	**1.27 (0.38)**	** < 0.001**
**FTF Internalizing symptoms**													
Screen time ≥ 75 pct	1.41	1.77	**0.36 (0.16)**	**0.030**	0.007	0.29 (0.17)	0.088	**0.33 (1.68)**	**0.050**	0.28 (0.16)	0.084	0.27 (0.16)	0.099
Depression ≥ 10 pts	1.39	2.14	**0.76 (0.17)**	** < 0.001**	0.029	**0.76 (0.18)**	** < 0.001**	–	–	–	–	0.28 (0.20)	0.159
Stress ≥ 11 pts	1.47	1.94	**0.47 (0.20)**	**0.017**	0.009	**–**	**–**	**0.56 (0.20)**	**0.005**	–	–	0.02 (0.22)	0.923
Parenting style dimension													
Low affection ≤ 25 pct	1.45	1.86	**0.41 (0.17)**	**0.014**	0.009	**–**	**–**	**–**	**–**	0.03 (1.18)	0.856	0.22 (0.18)	0.900
Behavioral control ≥ 75 pct	1.56	1.50	0.06 (1.61)	0.738	< 0.001	**–**	**–**	**–**	**–**	0.12 (0.16)	0.443	0.14 (0.16)	0.388
Parenting stress ≥ 75 pct	1.26	2.47	**1.21 (0.16)**	** < 0.001**	0.082	**–**	**–**	**–**	**–**	**1.31 (0.17)**	** < 0.001**	**1.23 (0.19)**	** < 0.001**
**FTF Externalizing symptoms**													
Screen time ≥ 75 pct	2.63	3.26	**0.63 (0.29)**	**0.031**	0.007	**0.59 (0.29)**	**0.046**	**0.59 (0.29)**	**0.022**	**0.57 (0.28)**	**0.035**	**0.56 (0.28)**	**0.049**
Depression ≥ 10 pts	2.54	4.08	**1.54 (0.31)**	** < 0.001**	0.036	**1.04 (0.36)**	** < 0.001**	–	–	–	–	**0.91 (0.35)**	**0.011**
Stress ≥ 11 pts	2.72	3.66	**0.93 (0.36)**	**0.010**	0.010	**–**	**–**	**1.68 (0.31)**	**0.004**	–	–	0.09 (0.38)	0.812
Parenting style dimension													
Low affection ≤ 25 pct	2.59	3.76	**1.16 (0.30)**	** < 0.001**	0.022	**–**	**–**	**–**	**–**	0.60 (0.28)	0.097	0.48 (0.31)	0.119
Behavioral control ≥ 75 pct	2.71	3.31	**0.60 (0.30)**	**0.044**	0.006	**–**	**–**	**–**	**–**	0.37 (0.28)	0.191	0.33 (0.28)	0.249
Parenting stress ≥ 75 pct	2.33	4.59	**2.25 (0.29)**	** < 0.001**	0.085	**–**	**–**	**–**	**–**	**2.15 (0.31)**	** < 0.001**	**1.87 (0.32)**	** < 0.001**

^a^Means of FTF symptoms in the low risk (below cutoff) and high risk (above cutoff) groups for the given dependent variable; for low affection low risk > cutoff, high risk < cutoff. Valid N’s 631-643

^b^ Screen time + Background factors + Maternal depression. Valid N’s 609-611

^c^ Screen time + Background factors + Maternal stress. Valid N’s 609-611

^d^ Screen time + Background factors + Maternal parenting style dimensions (low affection, high behavioral control, high parental stress). Valid N’s 609-611

^e^ Screen time + Background factors + Maternal depression + Maternal stress + Maternal parenting style dimensions (low affection, high behavioral control, high parental stress). Valid N’s 609-611

In the responses of fathers, we found in the first set of ANOVA models (i.e., univariate models) that higher levels of children’s screen time were associated with their attention difficulties as well as hyperactivity and impulsivity, (both ps < 0.001) (Table 3, univariate models). Related to child’s attention and concentration difficulties and hyperactivity and impulsivity, these associations remained significant when we controlled for depression, stress, or parenting style dimensions (Table 3, models 1–3), and also in the full model, when all factors were jointly studied (Table 3, models 4) (both ps ≤ 0.005), indicating that child’s screen time was associated with these symptoms independently, i.e., despite paternal distress and parenting style dimensions being taken into account. The associations of children’s screen time with internalizing and externalizing symptoms were not significant in these models. Of the paternal mental well-being and parenting style dimensions, in the final models, fathers’ behavioral control remained a significant predictor of children’s internalizing symptoms and parenting stress was a significant predictor of hyperactivity and externalizing symptoms (Table 3, models 4).

**Table 3:** The influence of fathers’ mental well-being and parenting style dimensions on the association between child’s screen time and psychosocial symptoms (Father’s *N* = 531)

	Low risk group^a^	High risk group^a^	Univariate			Model 1^b^		Model 2^c^		Model 3^d^		Model 4^e^	
	Mean	Mean	B (se)	p	partial eta^2^	B (se)	p	B (se)	p	B (se)	p	B (se)	p
**FTF Attention and concentration difficulties**													
Screen time ≥ 75 pct	3.33	4.72	**1.40 (0.37)**	** < 0.001**	0.032	**1.28 (0.37)**	** < 0.001**	**1.27 (0.37)**	** < 0.001**	**1.25 (0.37)**	** < 0.001**	**1.26 (0.37)**	** < 0.001**
Depression ≥ 10 pts	3.66	3.72	0.06 (0.47)	0.907	< 0.001	0.02 (0.46)	0.958	–	–	**–**	**–**	0.19 (0.51)	0.715
Stress ≥ 11 pts	3.65	3.82	0.17 (0.54)	0.750	< 0.001	**–**	**–**	0.15 (0.52)	0.769	**–**	**–**	0.04 (0.58)	0.950
Parenting style dimension													
Low affection ≤ 25 pct	3.42	4.30	**0.88 (0.36)**	**0.014**	0.013	**–**	**–**	**–**	**–**	0.56 (0.37)	0.128	0.57 (0.37)	0.125
Behavioral control ≥ 75 pct	3.62	3.83	0.21 (0.37)	0.571	0.001	**–**	**–**	**–**	**–**	0.24 (0.37)	0.514	0.25 (0.37)	0.500
Parenting stress ≥ 75 pct	3.49	4.20	0.70 (0.37)	0.062	0.008	**–**	**–**	**–**	**–**	0.38 (0.39)	0.325	0.42 (0.40)	0.292
**FTF Hyperactivity and impulsivity symptoms**													
Screen time ≥ 75 pct	3.58	4.95	**1.37 (0.40)**	** < 0.001**	0.027	**1.18 (0.39)**	**0.003**	**1.13 (0.39)**	**0.004**	**1.13 (0.39)**	**0.004**	**1.10 (0.40)**	**0.005**
Depression ≥ 10 pts	3.87	4.27	0.40 (0.50)	0.418	0.001	0.21 (0.49)	0.662	–	–	**–**	**–**	0.51 (0.54)	0.344
Stress ≥ 11 pts	3.81	4.95	**1.14 (0.56)**	**0.042**	0.009	**–**	**–**	**1.15 (0.56)**	**0.039**	**–**	**–**	0.96 (0.62)	0.120
Parenting style dimension													
Low affection ≤ 25 pct	3.62	4.71	**1.09 (0.37)**	**0.004**	0.018	**–**	**–**	**–**	**–**	0.55 (0.39)	0.155	0.51 (0.39)	0.196
Behavioral control ≥ 75 pct	3.86	4.11	0.25 (0.39)	0.532	0.001	**–**	**–**	**–**	**–**	0.40 (0.39)	0.310	0.38 (0.39)	0.333
Parenting stress ≥ 75 pct	3.62	4.86	**1.24 (0.39)**	**0.002**	0.022	**–**	**–**	**–**	**–**	**0.94 (0.41)**	**0.021**	**0.91 (0.42)**	**0.032**
**FTF Internalizing symptoms**													
Screen time ≥ 75 pct	1.43	1.62	0.20 (0.19)	0.298	0.002	0.19 (0.19)	0.330	0.17 (0.19)	0.365	0.21 (0.19)	0.274	0.20 (0.19)	0.299
Depression ≥ 10 pts	1.49	1.68	0.20 (0.24)	0.403	0.002	0.20 (0.24)	0.398	**–**	**–**	**–**	**–**	0.01 (0.26)	0.984
Stress ≥ 11 pts	1.47	1.85	0.37 (0.27)	0.166	0.004	**–**	**–**	0.39 (0.27)	0.149	**–**	**–**	0.25 (0.30)	0.412
Parenting style dimension													
Low affection ≤ 25 pct	1.42	1.75	0.33 (0.18)	0.070	0.007	**–**	**–**	**–**	**–**	0.29 (0.19)	0.123	0.27 (0.19)	0.150
Behavioral control ≥ 75 pct	1.39	1.89	**0.51 (0.19)**	**0.007**	0.016	**–**	**–**	**–**	**–**	**0.55 (0.19)**	**0.004**	**0.54 (0.19)**	**0.005**
Parenting stress ≥ 75 pct	1.43	1.76	0.33 (0.19)	0.081	0.007	**–**	**–**	**–**	**–**	0.21 (0.20)	0.283	0.18 (0.20)	0.380
**FTF Externalizing symptoms**													
Screen time ≥ 75 pct	2.63	3.22	0.59 (0.35)	0.092	0.006	0.48 (0.35)	0.175	0.43 (0.35)	0.218	0.43 (0.35)	0.219	0.40 (0.35)	0.258
Depression ≥ 10 pts	2.74	3.43	0.70 (0.45)	0.125	0.005	0.56 (0.44)	0.203	**–**	**–**	**–**	**–**	0.17 (0.48)	0.725
Stress ≥ 11 pts	2.72	3.82	**1.10 (0.51)**	**0.032**	0.010	–	–	**1.27 (0.50)**	**0.011**	**–**	**–**	0.92 (0.55)	0.096
Parenting style dimension													
Low affection ≤ 25 pct	2.56	3.56	**1.00 (0.34)**	**0.004**	0.019	**–**	**–**	**–**	**–**	0.40 (0.35)	0.245	0.35 (0.35)	0.321
Behavioral control ≥ 75 pct	2.73	3.17	0.44 (0.36)	0.223	0.003	**–**	**–**	**–**	**–**	0.28 (0.35)	0.427	0.25 (0.35)	0.479
Parenting stress ≥ 75 pct	2.49	3.93	**1.44 (0.35)**	** < 0.001**	0.036	**–**	**–**	**–**	**–**	**1.14 (0.36)**	**0.002**	**1.05 (0.38)**	**0.005**

^a^Means of FTF symptoms in the low risk (below cutoff) and high risk (above cutoff) groups for the given dependent variable; for low affection low risk > cutoff, high risk < cutoff. Valid N’s 437-454

^b^ Screen time + Background factors + Paternal depression. Valid N’s 434-436

^c^ Screen time + Background factors + Paternal stress. Valid N’s 434-436

^d^ Screen time + Background factors + Paternal parenting style dimensions (low affection, high behavioral control, high parental stress). Valid N’s 434-436

^e^ Screen time + Background factors + Paternal depression + Paternal stress + Paternal parenting style dimensions (low affection, high behavioral control, high parental stress), Valid N’s 433-435

**Fig. 1:**
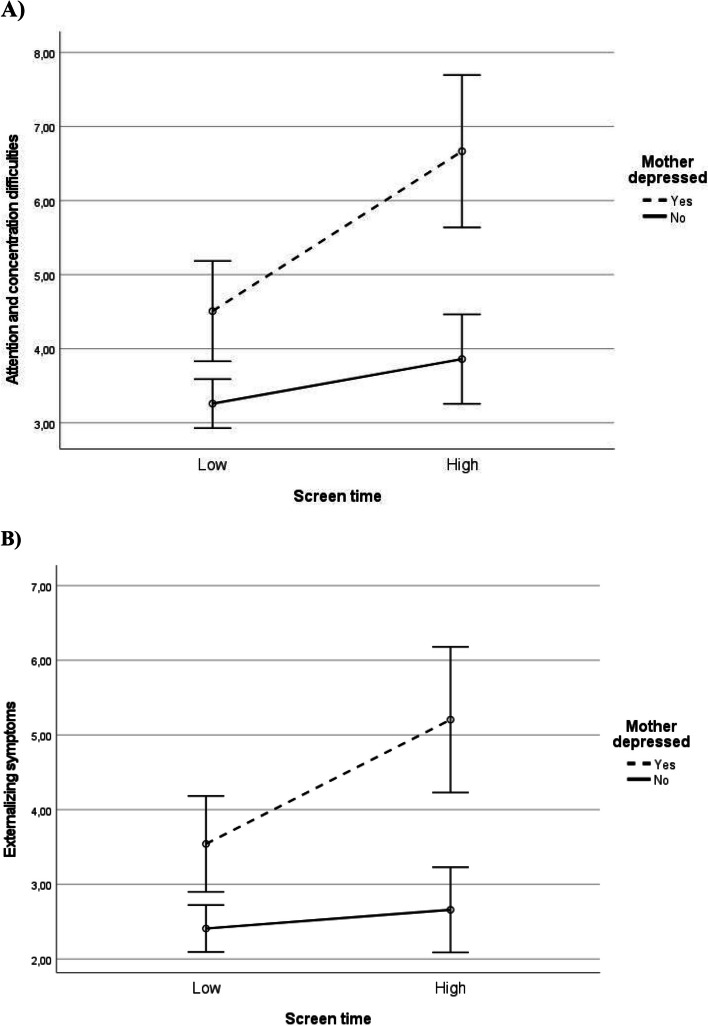
Associations of child’s screen time with attention and concentration difficulties (**A**) and with externalizing symptoms (**B**) as moderated by mother’s depression. Estimated marginal means from ANOVA models. Error bars represent 95% confidence intervals

**Fig. 2:**
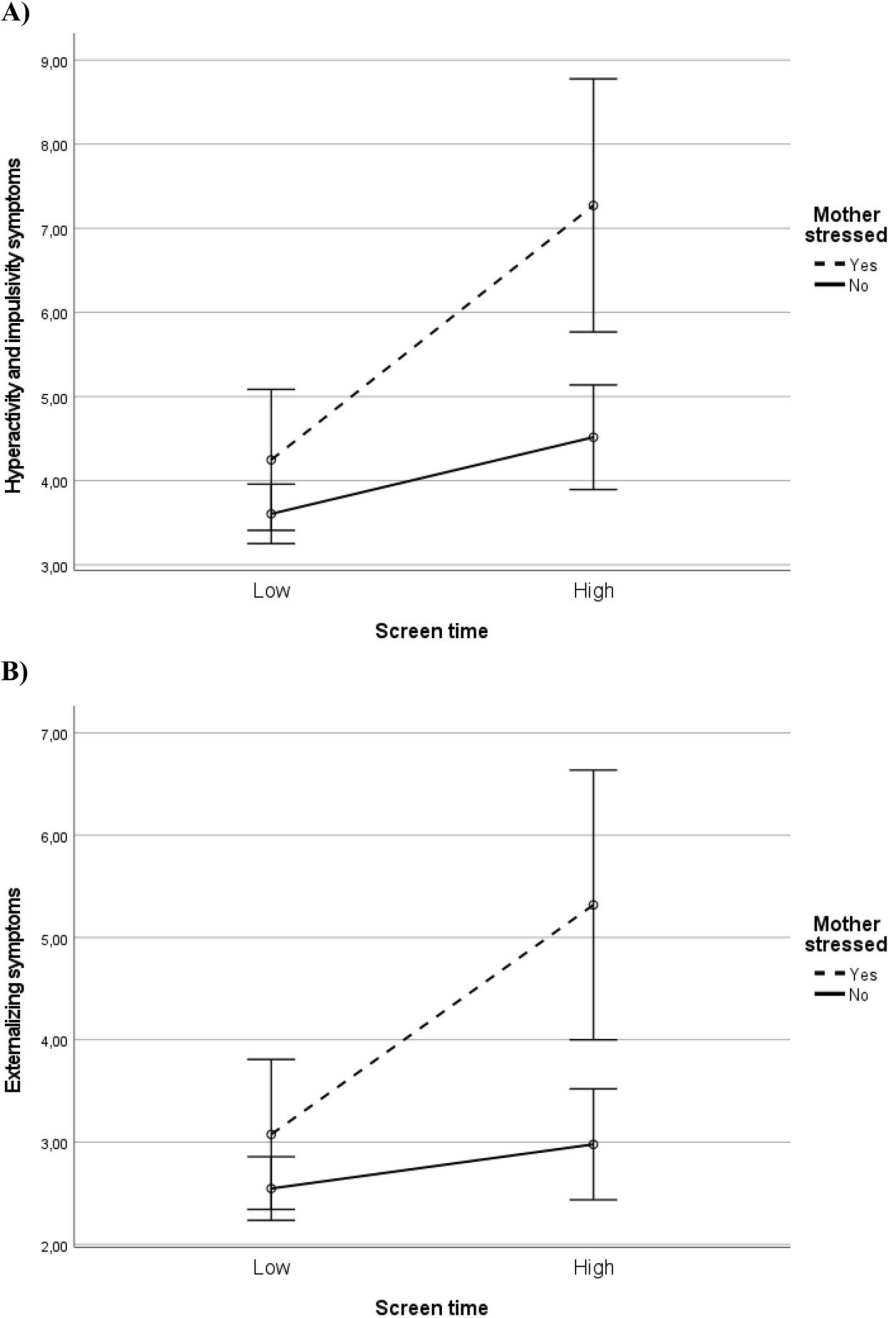
Associations of child’s screen time with hyperactivity and impulsivity symptoms (**A**) and with externalizing symptoms (**B**) as moderated by mother’s stress. Estimated marginal means from ANOVA models. Error bars represent 95% confidence intervals

In moderation analyses among mothers, interaction terms between children’s screen time and maternal depression were significant on attention and concentration difficulties (*p* = 0.031) and externalizing symptoms (*p* = 0.038) in the unadjusted models. Similarly, interaction terms between children’s screen time and their mother’s stress were significant with regard to the children’s hyperactivity and impulsivity (*p* = 0.026) and externalizing symptoms (*p* = 0.030) in the unadjusted models. Regarding all these significant interactions, the association between child’s screen time and psychosocial symptoms was more pronounced when the mother was depressed or stressed, while in the case of a non-depressed or non-stressed mother the association was weaker or absent (Figs. 1 and 2). The interaction terms between child’s screen time and mother’s depression attenuated after controlling for background factors and turned non-significant (*p* > 0.05), while the interaction terms regarding mother’s stress remained significant in the adjusted models. There were no significant interaction terms regarding mother’s parenting style dimensions. Among fathers, there were no significant interactions regarding any of the five studied moderators (parental depression, stress and the three parenting style dimensions). Supplementary moderation analyses using continuous variables are shown in the Appendix 2 (supplementary material).

## Discussion

In this study, our aim was to examine whether the association between a child’s screen time and psychosocial symptoms is in fact explained by parent-related factors, including parental depression and stress and parenting style dimensions. Our results indicate that for the most part it is not. That is, the associations between children’s higher screen time and higher attention and concentration difficulties as well as hyperactivity and impulsivity symptoms and externalizing symptoms remained significant even after controlling for parents’ mental health, their parenting style dimensions as well as multiple background factors. However, the association between screen time and internalizing symptoms did not remain significant. Thus, among mothers, family-level factors seem to attenuate the association between screentime and internalizing symptoms, while the effect was relatively weak to begin with (*p* = 0.030). These findings seem to suggest that the risks attributed to screen time do not merely reflect parent-related distress and family adversity, but instead the screen time is independently associated with child’s mental health when parent-related distress and family adversity are statistically taken into account.

In addition to the independent role of screen time on child’s psychosocial symptoms, we further analyzed whether these associations are moderated by parent-related factors. These analyses showed that children are reported to have more psychosocial symptoms when their mothers are depressed/stressed and they are exposed to more screentime, compared to other children.

Consistently with our earlier findings [3], screen time was associated with higher levels of children’s psychosocial symptoms. This result is in line with other studies that have found an association between children’s screen time and psychosocial symptoms, such as internalizing and externalizing problems, as well as inattention and impulsivity [6–10, 30]. As these studies have not controlled for parenting style dimensions or parental stress, our findings add to the literature by showing that the associations remain significant, even when these factors are taken into account. Our results especially emphasize how screen time is related to attention and concentration difficulties, hyperactivity and impulsivity symptoms, as well as externalizing problems regardless of psychological distress in the family.

There are multiple mechanisms that could explain the results. In terms of hyperactivity and impulsivity as well as concentration problems, previous studies suggest that screen time may prohibit their availability for activities that are considered to enhance cognitive capacities and stimulate longer attention span [31–33]. Interestingly, also in our study, associations between screen time and hyperactivity and concentration problems were the most robust ones, prevailing as significant in the final adjusted models. It has also been suggested that parents in less affluent families would be more prone to use mobile technology to calm their children or keep them quiet, particularly parents who express lower perceived control over their children’s behavior and development [34]. However, in our study, most of the parents were well-educated. Furthermore, all associations between children’s screen time and psychosocial problems remained significant when we controlled for parental education. This again emphasizes the point that the risks related to children’s screen time cannot solely be reduced to family-level factors. It is worthy of note that the sample comprises generally typically developing children from a birth cohort, wherein children with diagnosed neurobehavioral disorders were excluded. Therefore, the findings are limited to mainly typically developing children and suggest that even in such a normative sample, symptoms of impulsivity and inattention can be related to screentime. For example, children with neuropsychiatric disorders (e.g., ADHD) may be predisposed to more electronic media use. They may, for example, have more difficulties to quit playing. We propose that children with ADHD should be studied in clinical samples, not in birth cohorts like the present study to ensure adequate sample size.

Our results illustrated that risks related to screen time and children’s psychosocial symptoms were moderated by maternal depression and stress. Although previous studies have found an association between maternal depressive symptoms and children’s increased screen time [15], they have not reported how a mother’s stress or depression moderates the association between screen time and their child’s symptoms. However, Huesmann & Taylor [35] found that exposure to violent media content is associated with externalizing symptoms such as aggressive behavior, but active discussion between parents and children when exposed to aggressive material may buffer the effect. Moreover, Fam et al. [36] found that parents’ active mediation (i.e., communication with a child and discussion about media-related concerns) was negatively associated with problematic e-media use. Moreover, studies have found that parents’ passive co-viewing of programs with a child is detrimental to children’s psychosocial well-being [36, 37]. It may be that when a mother is depressed or stressed, she may not be able to actively participate in her child’s e-media use (discussions, parental mediation) and thus not be able to buffer the negative effects between the child’s increased screen time and psychosocial symptoms.

Our results showed that for the most, parenting style dimensions did not significantly moderate the association between children’s screen time and psychosocial symptoms. This differs from some previous studies reporting that inconsistent parenting and problems in parent–child interaction have an additional negative effect on the association between a preschooler’s screen time and psychosocial well-being [13, 14]. Also in our study, parenting style dimensions characterized by low affection, high behavioral control or high parenting stress were associated with higher levels of children’s psychosocial symptoms among both mothers and fathers (see also [38]), and in fact parenting stress had the strongest effects on child’s psychosocial symptoms, especially among mothers. However, with one exception, the effect of maternal parenting stress on child internalizing symptoms (see Appendix 2), the parenting style dimensions did not moderate the association between screen time and child outcomes. Among fathers, none of the studied moderators of parental distress and parenting styles significantly moderated the association between children’s increased screen time and psychosocial symptoms. This may be due to a smaller number of fathers participating in our study and thus a loss of power to detect interactions. Alternatively, the mechanisms relating to children’s screen time and psychosocial problems may be different between mothers and fathers. More studies are needed to further examine these issues among fathers.

The results of the current study are not caused by parental distress being correlated with the child’s screentime because child’s screentime was not associated with parental distress (Appendix 1) except for maternal affection. Nevertheless, we point out that the associations between the factors are complex and—as such—interpretations of the causality cannot be made. We instead argue that the link between the factors might be bidirectional. For example, it is possible that the child’s behavioral problems could be related to higher amount of screen time. This is supported by a study of Ansari et al. [16] which shows that preschooler’s hyperactive behavior increased their screen viewing over time (see also Radesky et al. [34]). Moreover, parents’ behavior and psychological well-being might be related to screen time with multiple ways – for example some parents might use e-media devices as a tool to calm their children down, especially among children with socio-emotional difficulties. Furthermore, there is a possibility that stressed mothers allow more screen time to cope with the child’s problem behaviors. For example, Radesky et al. [34] argue that when parents do not have control over the child’s behavior it might cause both the stress and allowance of child’s extensive media use. One example of complex relationship between the studied factors (appearing also in our study) is a work by Parks et al. [42] where authors noticed that parents who expressed not wanting their stress to affect their child were the same parents that allowed their child to watch television. They considered that this allowed them to control their emotions and not show negative feelings towards their child.

As a limitation of our study, we mention that those with lower educational achievement are under-represented in the sample, which seems to be common according to studies on drop-out rates in longitudinal studies on mental health [39]. Within the participating parents, however, we controlled the models for socio-economic status to take its effect into account. Moreover, we emphasize that missingness in this data was not random—it was related to several well-known stress factors in the families, such as depression, lower education, and to higher number of children. As these factors are usually associated with more psychosocial symptoms in children, drop out in this study might lead to underestimated risk estimates relative to the entire target population. Hence, the results presented here can not necessarily be generalized to entire population, but the less risky proportion of the target population. As another limitation we mention that in survey studies, such as ours, it may be difficult for parents to evaluate their children’s screen time precisely. Moreover, this study is a cross-sectional birth cohort study, and as such we cannot make interpretations of the causality. Further, we cannot rule out the possibility of reverse causality (that for example attention problems lead to more screen time) or the influence of other variables such as genetic dispositions. Future studies should longitudinally evaluate the symptoms trajectories and how they relate to screentime at different timepoints. Moreover, we did not measure parental stress on their children’s screentime. However, we found that parental depression and stress were not correlated with the child’s screentime in this study. Despite this, in the future, more studies are needed to evaluate the effect of parents’ stress on their children’s screen time.

It should be noted, that e-media content and parenting practices during the viewing could moderate the risks related to screen time [37]. In the current study, we were not able to control the age appropriateness of the screen content nor the parenting practices or mediation the screen time. Finally, stress factors tend to have a cumulative impact on children; the more children are exposed to various risk factors, the higher their risk [38]. Further studies are therefore needed to evaluate whether there is a cumulative effect of screen time and other stress factors relative to children’s symptoms.

## Conclusions

Among young children, a high amount of screen time is independently related to multiple psychosocial symptoms even when various factors related to parents’ mental health and parenting style dimensions have been taken into account. In addition, the risk seems to be especially pronounced when a mother is stressed or depressed. Various psychosocial environmental risk factors in childhood (interacting with genetic dispositions) play a cumulative role relative to children’s well-being making it important to study multiple factors simultaneously [40, 41]. It is also important to keep in mind that the relationship between these factors may be bidirectional. In clinical practice, the length of children’s screen time should be inquired already at a young age. Furthermore, parents should be offered guidance to reduce the possible ill effects of excessive screen time and help with their own mental health problems. Recognizing the risk factors related to excessive screen time and parents’ mental health is crucial when children are young to prevent psychosocial symptoms from developing further. Moreover, monitoring these issues when children grow older, is equally important. As screen use is increasingly a universal part of children’s daily lives, intervention studies are needed that aim to reduce children’s psychosocial symptoms related to higher levels of screen time. Enhancing parents’ active mediation and discussion of children’s e-media habits in families can potentially prove to be promising avenues for these efforts.

### Supplementary Information

Supplementary Material 1.

Supplementary Material 2.

## Data Availability

The data of this study is not publicly available due to legal restrictions and confidential nature. The data is available upon request. Requests may be sent to The Finnish Institute for Health and Welfare, who is the controller of the data. Contact information: juulia.paavonen@helsinki.fi.
