# Supplementary material for: Children’s screen time and psychosocial symptoms at 5 years of age – the role of parental factors
Source: BMC Pediatr. 2024 Aug 3;24:500. doi: 10.1186/s12887-024-04915-8 (PMC11297624; doi:10.1186/s12887-024-04915-8)
Supplement: Supplementary file 1 — Supplementary Material 1. [file 12887_2024_4915_MOESM1_ESM.docx]

**Appendix 1.** Correlations between the study variables; Pearson correlation coefficient.

|  | Screen  time | FTF Attention and  concentration difficulties | FTF  Hyperactivity and impulsivity symptoms | FTF Internalizing symptoms | FTF Externalizing symptoms | Depression. mothers | Stress.  mothers | Parenting style dimensions.  Affection. mother | Parenting style dimensions.  behavioral control. mother | Parenting style dimensions.  parenting stress. mother | Depression. fathers | Stress.  fathers | Parenting style dimensions.  affection. father | Parenting style dimensions.  behavioral control. father | Parenting style dimensions.  parenting stress. father |
| --- | --- | --- | --- | --- | --- | --- | --- | --- | --- | --- | --- | --- | --- | --- | --- |
| Screen time | 1 | 0.199** | 0.146** | 0.121** | 0.130** | 0.067 | 0.039 | -0.100* | 0.034 | 0.044 | 0.019 | 0.007 | -0.068 | -0.044 | 0.047 |
| FTF Attention and concentration difficulties |  | 1 | 0.601** | 0.338** | 0.498** | 0.251** | 0.255** | -0.201** | 0.134** | 0.283** | 0.034 | 0.055 | -0.116* | 0.053 | 0.100* |
| FTF Hyperactivity and impulsivity symptoms |  |  | 1 | 0.281** | 0.505** | 0.198** | 0.176** | -0.179** | 0.081* | 0.247** | 0.064 | 0.078 | -.121** | 0.083 | 0.151** |
| FTF Internalizing symptoms |  |  |  | 1 | 0.480** | 0.276** | 0.167** | -0.134** | -0.007 | 0.321** | 0.067 | 0.084 | -0.025 | .110* | 0.102* |
| FTF Externalizing symptoms |  |  |  |  | 1 | 0.265** | 0.187** | -0.206** | 0.082* | 0.349** | 0.131** | 0.129** | -.125** | .105* | 0.182** |
| Depression. mothers |  |  |  |  |  | 1 | 0.665** | -0.230** | 0.001 | 0.468** | 0.185** | 0.142** | -0.033 | -0.024 | 0.101* |
| Stress. mothers |  |  |  |  |  |  | 1 | -0.181** | 0.071 | 0.463** | 0.175** | 0.183** | -0.012 | 0.057 | 0.138** |
| Parenting style dimensions. Affection. mother |  |  |  |  |  |  |  | 1 | -0.061 | -0.497** | -0.02 | -0.064 | .170** | -.109* | -0.215** |
| Parenting style dimensions. behavioral control. mother |  |  |  |  |  |  |  |  | 1 | 0.084* | -0.067 | -0.026 | -0.038 | .303** | -0.002 |
| Parenting style dimensions. parenting stress. mother |  |  |  |  |  |  |  |  |  | 1 | 0.108* | 0.133** | -.118* | 0.091 | 0.243** |
| Depression. fathers |  |  |  |  |  |  |  |  |  |  | 1 | 0.622** | -.252** | 0.005 | 0.432** |
| Stress. fathers |  |  |  |  |  |  |  |  |  |  |  | 1 | -.239** | -0.005 | 0.453** |
| Parenting style dimensions. Affection. father |  |  |  |  |  |  |  |  |  |  |  |  | 1 | 0.011 | -0.497** |
| Parenting style dimensions. behavioral control. father |  |  |  |  |  |  |  |  |  |  |  |  |  | 1 | 0.064 |
| Parenting style dimensions. parenting stress. father |  |  |  |  |  |  |  |  |  |  |  |  |  |  | 1 |

* Correlation is significant at the 0.05 level (2-tailed).

** Correlation is significant at the 0.01 level (2-tailed).

N mothers 620-670; N fathers 437-455
