# Supplementary material for: Children’s screen time and psychosocial symptoms at 5 years of age – the role of parental factors
Source: BMC Pediatr. 2024 Aug 3;24:500. doi: 10.1186/s12887-024-04915-8 (PMC11297624; doi:10.1186/s12887-024-04915-8)

**Appendix 2.** Supplementary moderation analyses using continuous measures. Only significant (p<0.05) moderations presented.


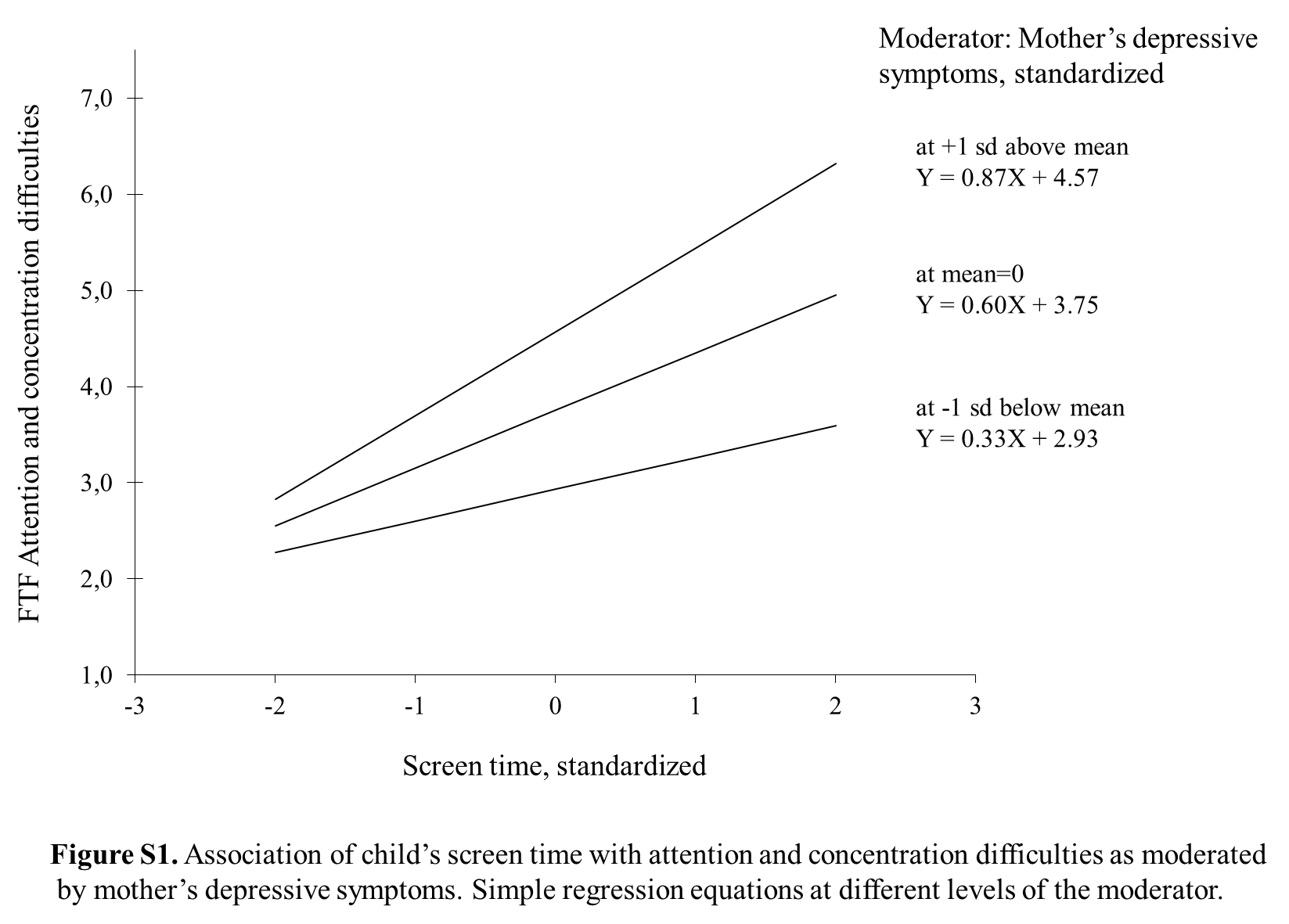


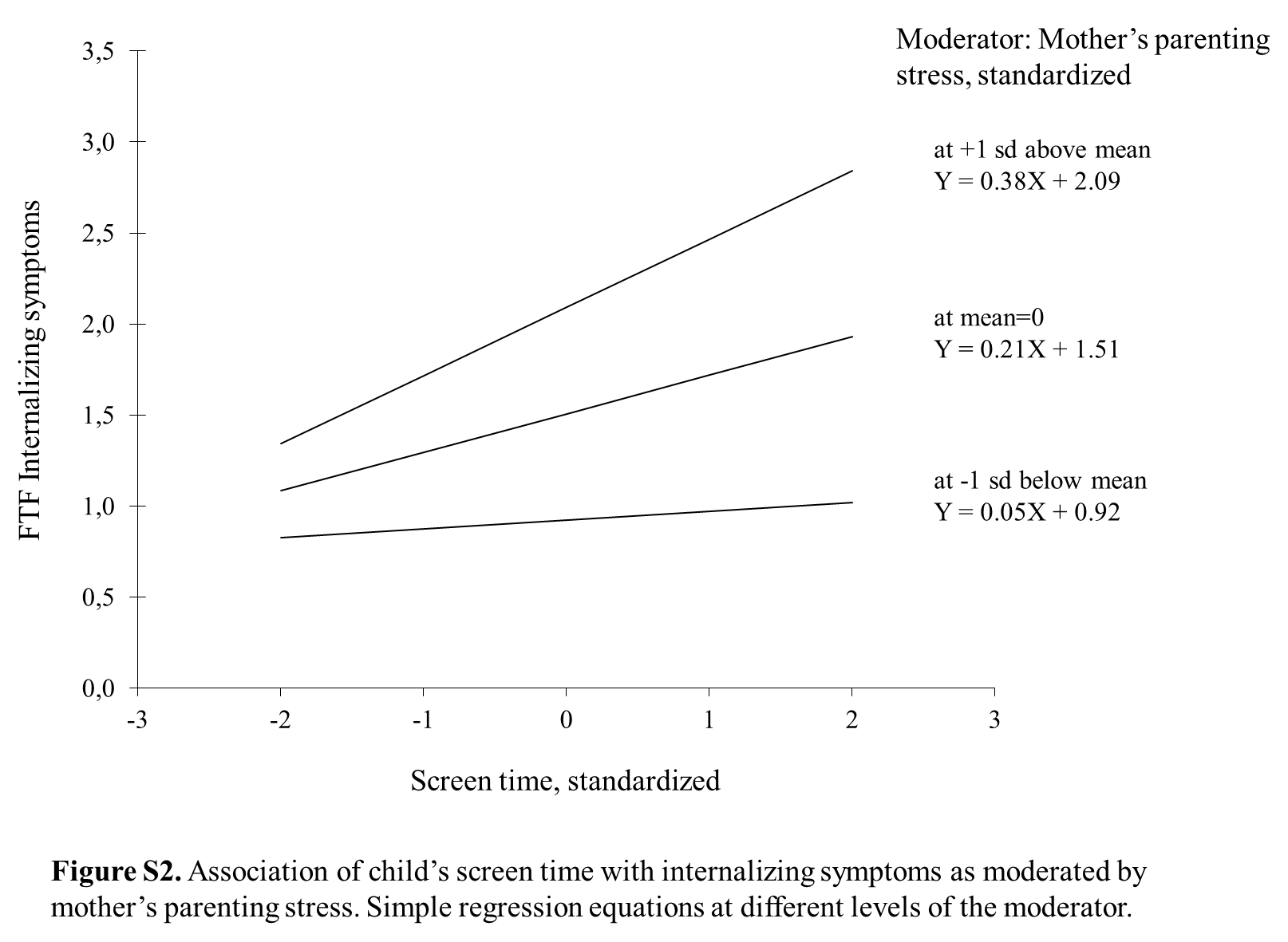

Supplement: Supplementary file 2 — Supplementary Material 2. [file 12887_2024_4915_MOESM2_ESM.docx]
